# Tuberculosis and other opportunistic infections in tofacitinib-treated patients with rheumatoid arthritis

**DOI:** 10.1136/annrheumdis-2015-207319

**Published:** 2015-08-28

**Authors:** K L Winthrop, S-H Park, A Gul, M H Cardiel, J J Gomez-Reino, Y Tanaka, K Kwok, T Lukic, E Mortensen, D Ponce de Leon, R Riese, H Valdez

**Affiliations:** 1 Oregon Health and Science University, Portland, Oregon, USA; 2 Division of Rheumatology, Department of Internal Medicine, Seoul St. Mary's Hospital, The Catholic University of Korea, Seoul, South Korea; 3 Istanbul Faculty of Medicine, Istanbul University, Istanbul, Turkey; 4 Centro de Investigación Clínica de Morelia SC, Morelia, Mexico; 5 Hospital Clínico Universitario de Santiago, Santiago de Compostela, Spain; 6 University of Occupational and Environmental Health, Kitakyushu, Japan; 7 Pfizer Inc, New York, New York, USA; 8 Pfizer Inc, Collegeville, Pennsylvania, USA; 9 Pfizer Inc, Lima, Peru; 10 Pfizer Inc, Groton, Connecticut, USA

**Keywords:** Tuberculosis, Infections, Treatment, Rheumatoid Arthritis, DMARDs (synthetic)

## Abstract

**Objectives:**

To evaluate the risk of opportunistic infections (OIs) in patients with rheumatoid arthritis (RA) treated with tofacitinib.

**Methods:**

Phase II, III and long-term extension clinical trial data (April 2013 data-cut) from the tofacitinib RA programme were reviewed. OIs defined a priori included mycobacterial and fungal infections, multidermatomal herpes zoster and other viral infections associated with immunosuppression. For OIs, we calculated crude incidence rates (IRs; per 100 patient-years (95% CI)); for tuberculosis (TB) specifically, we calculated rates stratified by patient enrolment region according to background TB IR (per 100 patient-years): low (≤0.01), medium (>0.01 to ≤0.05) and high (>0.05).

**Results:**

We identified 60 OIs among 5671 subjects; all occurred among tofacitinib-treated patients. TB (crude IR 0.21, 95% CI of (0.14 to 0.30)) was the most common OI (n=26); median time between drug start and diagnosis was 64 weeks (range 15–161 weeks). Twenty-one cases (81%) occurred in countries with high background TB IR, and the rate varied with regional background TB IR: low 0.02 (0.003 to 0.15), medium 0.08 (0.03 to 0.21) and high 0.75 (0.49 to 1.15). In Phase III studies, 263 patients diagnosed with latent TB infection were treated with isoniazid and tofacitinib concurrently; none developed TB. For OIs other than TB, 34 events were reported (crude IR 0.25 (95% CI 0.18 to 0.36)).

**Conclusions:**

Within the global tofacitinib RA development programme, TB was the most common OI reported but was rare in regions of low and medium TB incidence. Patients who screen positive for latent TB can be treated with isoniazid during tofacitinib therapy.

## Introduction

Tuberculosis (TB) and other opportunistic infections (OIs) occur more frequently in patients with rheumatoid arthritis (RA), and this risk is elevated by the use of prednisone and certain biological disease-modifying antirheumatic drugs (DMARDs).^1–3^ This has been best documented for granuloma-inducing pathogens in the setting of tumour necrosis factor-alpha (TNF) blockade.^4–9^ For biological therapies with other mechanisms of action, and for small molecular therapies like tofacitinib, less is known. Tofacitinib is a small-molecule oral Janus kinase (JAK) inhibitor approved for the treatment of adult patients with RA.^10^ Tofacitinib preferentially inhibits JAK3 and JAK1, modulating the immune response via downregulation of several cytokines (eg, interleukins (ILs) 2, 4, 7, 9, 15 and 21) that are integral to lymphocyte development and function.^11^ Given its mechanism of action, the risk of TB and other OIs could potentially be elevated in tofacitinib-treated patients, and accordingly, we undertook a retrospective evaluation of all OIs reported within the tofacitinib RA clinical development programme.

## Methods

### Development programme conduct

Following completion of phase I studies, the global tofacitinib RA development programme comprised six phase II,^12–17^ six phase III^18–23^ and two open-label long-term extension (LTE) studies,^24^ ^25^ with a total of 5671 treated patients and 12 664 patient-years tofacitinib exposure across 48 nations worldwide.

### OI case-finding and case description

For phase II, III and open-label LTE studies, we searched all preferred and low-level MedDRA terms (see online supplementary file 1) reported by site investigators containing text consistent with a potential OI using a data cut-off date of 10 April 2013. As of April 2013, data collection for one phase III study^23^ and the two LTE studies^24^ ^25^ were ongoing, and study databases had not yet been locked. For this analysis, the following infections were defined a priori as OIs: TB, non-tuberculous mycobacterium (NTM) infections, all fungal infections (with the exception of oral or vaginal candidiasis and chromomycosis), listeria and viral infections typically associated with immunosuppression, including multidermatomal or disseminated herpes zoster, disseminated herpes virus, cytomegalovirus (CMV), BK virus and progressive multifocal leukoencephalopathy (PML). Legionella was not specified as an OI and was not included in this analysis (one case was reported during the development programme).

For cases classified as ‘OIs’, we reviewed adverse reaction report forms to obtain clinical details, including treatment and outcomes. For all cases, we collected descriptive clinical and epidemiological information at time of randomisation (baseline) including age, sex, race, site of enrolment, corticosteroid use (limited by protocol to ≤10 mg/day prednisone equivalent), concomitant non-biological DMARD use, comorbidities (eg, smoking, diabetes, others), body mass index, measures of RA severity (Disease Activity Score using 28-joint counts and the C reactive protein level (DAS 28-3 CRP)) and disease duration (years since diagnosis), as well as baseline lymphocyte and neutrophil counts.

### TB screening

In phase II studies, patients with positive TB screening were excluded from entering the trial. In phase III studies, all potential study subjects were queried regarding a history of prior diagnosis or treatment of active or latent TB infection (LTBI). All patients underwent chest radiography (within 3 months of screening), and for those with no prior history of positive screening results, at least one screening test for LTBI was performed at discretion of the site primary investigator; QuantiFERON-TB Gold In-Tube (QFT-IT; Quest Diagnostics, Madison, New Jersey, USA) or tuberculin skin test (TST; using ≥5 mm induration cut-off outside Japan and ≥20 mm erythema for Japan). Patients diagnosed with LTBI and patients with a history of inadequately or untreated LTBI prior to screening were allowed entry into phase III trials after receiving at least 1 month of a planned 9-month isoniazid preventive therapy regimen.

### OI incidence rate calculations and evaluation of comparative risk

OIs were attributed either to tofacitinib, placebo, adalimumab or methotrexate based on exposure at time of event. Given all OIs occurred in tofacitinib patients, we calculated OI crude incidence rates (IRs) per 100 patient-years (95% CI) only for tofacitinib-exposed patients. Given that no OIs occurred in phase II studies, these calculations were limited to the phase III and LTE studies. Patients were censored at time of event, death or withdrawal from study.

### TB substudy

For TB cases, we obtained detailed information regarding date of onset, type of presentation and clinical outcomes. We also evaluated TB screening results among patients who entered the phase II, III and LTE studies. In addition, for patients who started isoniazid preventive therapy and entered trials, we described the proportion of individuals who developed elevated transaminases. We calculated crude TB IRs overall, as well as stratified by enrolment region, age, sex and baseline corticosteroid use. In order to contextualise the regional variation in TB rates, we presented TB rates according to regional background TB incidence. We used WHO TB incidence data from 2011,^26^ to categorise countries where enrolment took place according to background TB IR (per 100 patient-years) defined as follows: low-incidence regions (IR≤0.01 per 100 patient-years), medium-incidence regions (IR>0.01 to ≤0.05 per 100 patient-years) and high-incidence regions (IR >0.05 per 100 patient-years). We conducted all statistical analyses using SAS software (SAS Institute, Cary, North Carolina, USA).

## Results

Patient baseline characteristics are described in table 1. We identified 60 OIs (58 patients) among 5671 patients enrolled in phase II, phase III and LTE studies (crude IR (95% CI) of 0.46 (0.36 to 0.59) per 100 patient-years); no events occurred during phase II studies and all events occurred in tofacitinib-treated patients. Crude IRs for TB and non-TB OIs were 0.21 (95% CI 0.14 to 0.30) and 0.25 (95% CI 0.18 to 0.36), respectively. OIs included TB (n=26), oesophageal candidiasis (n=9), *Pneumocystis jirovecii* pneumonia (n=4), CMV infection (n=6), NTM pulmonary infection (n=2), cryptococcal infection (pneumonia n=2, meningitis n=1), disseminated or multidermatomal herpes zoster (n=8), BK encephalopathy (n=1) and toxoplasmosis (n=1). No cases of disseminated herpes virus or PML were reported. Of the 58 patients with OIs, one patient died due to pneumocystis, and most (n=40) permanently discontinued treatment with the study drug. The CMV cases presented differently: antigenemia without evidence of other infection (n=1); an oesophageal ulcer, which resolved without antiviral therapy while tofacitinib treatment was continued (n=1); sialadenitis with CMV on biopsy (n=1); hepatitis in which CMV was also detected in cerebrospinal fluid (CSF) by PCR (n=1); and gastritis with little clinical information provided (n=1). The final case involved CMV retinitis with characteristic retinal pathology and a positive anterior chamber PCR; the infection responded appropriately to antiviral therapy. The case of BK encephalitis was diagnosed using PCR of CSF in a patient during an episode of bacterial sepsis; it resolved as the patient's overall status improved.

**Table 1:** Baseline characteristics of patients entering phase III tofacitinib trials by exposure group

	Tofacitinib 5 mg twice daily (N=1587; PY=1464.18)	Tofacitinib 10 mg twice daily (N=1609; PY=1501.03)	Placebo (N=681; PY=202.71)	Adalimumab (N=204; PY=178.04)	Methotrexate (N=186; PY=152.07)
Age, median years (range)	54 (18–86)	54 (18–86)	54 (18–82)	54 (24–78)	50.5 (20–80)
Female, n (%)	1310 (82.5)	1355 (84.2)	553 (81.2)	162 (79.4)	145 (78.0)
Race, n (%)					
White	976 (61.5)	1006 (62.5)	439 (64.5)	148 (72.5)	127 (68.3)
Black	58 (3.7)	47 (2.9)	24 (3.5)	3 (1.5)	4 (2.2)
Asian	394 (24.8)	375 (23.3)	166 (24.4)	29 (14.2)	33 (17.7)
Other	159 (10)	181 (11.2)	52 (7.6)	24 (11.8)	22 (11.8)
RA duration, mean, years	7.4	7.7	9.3	8.1	2.6
Diabetes mellitus, n (%)	130 (8.2)	127 (7.9)	48 (7.0)	16 (7.8)	8 (4.3)
COPD, n (%)	125 (7.9)	134 (8.3)	64 (9.4)	11 (5.4)	9 (4.8)
Smoking history, n (%)	534 (33.7)	520 (32.3)	254 (37.4)	71 (35.1)	61 (32.8)
BMI, mean (range)	26.9 (14.3–70.8)	27.0 (12.1–63.3)	27.2 (14.7–55.1)	27.1 (13.9–45.7)	26.7 (14.9–49.4)
RA severity (DAS28-3 CRP)	5.4	5.4	5.3	5.3	5.6
Concomitant DMARD, n (%)					
Methotrexate	904 (57.0)	902 (56.1)	520 (76.4)	199 (97.5)	1 (0.5)
Leflunomide	91 (5.7)	84 (5.2)	34 (5.0)	0 (0)	0 (0)
Hydroxychloroquine	152 (9.6)	157 (9.8)	51 (7.5)	2 (<1.0)	26 (14.0)
Baseline glucocorticoid use, n (%)	866 (54.6)	834 (51.8)	376 (55.2)	116 (56.9)	79 (42.5)
>0 mg to <5 mg daily	114 (7.2)	113 (7.0)	48 (7.0)	16 (7.8)	10 (5.4)
5 to 10 mg daily	731 (46.1)	698 (43.4)	315 (46.3)	90 (44.1)	68 (36.6)
>10 mg daily	9 (<1.0)	9 (<1.0)	5 (<1.0)	7 (3.4)	0 (0)
Unknown dose	24 (1.5)	20 (1.2)	19 (2.8)	3 (1.5)	3 (1.6)

BMI, body mass index; COPD, chronic obstructive pulmonary disease; DAS28-3 CRP, Disease Activity Score using 28-joint counts and the C reactive protein level; DMARD, disease-modifying antirheumatic drugs; PY, person-years exposure; RA, rheumatoid arthritis.

### All OIs (TB and non-TB OIs combined)

In phase III studies, crude OI incidence was numerically higher in patients treated with tofacitinib 10 mg twice daily (0.93 (95% CI 0.55 to 1.58)) vs 5 mg twice daily (0.20 (95% CI 0.07 to 0.64)). In LTE studies, rates were more similar in tofacitinib 5 mg (0.38 (95% CI 0.23 to 0.62)) and 10 mg treated patients (0.48 (95% CI 0.33 to 0.71)). Across treatment arms, the crude incidence of all OIs among all tofacitinib users was slightly higher among glucocorticoid users (0.55 (95% CI 0.38 to 0.81)) than non-users (0.31 (95% CI 0.19 to 0.53)). Rates were similar in those aged ≥65 and <65 years (0.49 (95% CI 0.24 to 1.04) vs 0.43 (95% CI 0.30 to 0.60)), respectively.

### OIs other than TB

Non-TB OIs occurred at a median of 40 weeks (range, 6–179 weeks) after tofacitinib start. In phase III studies, the incidence of non-TB OIs was numerically higher in patients treated with tofacitinib 10 mg twice daily (0.40 (95% CI 0.18 to 0.90) compared with 5 mg twice daily (0.21 (95% CI 0.07 to 0.64)). In LTE studies, rates were similar in tofacitinib 5 mg (0.23 (95% CI 0.12 to 0.43)) and 10 mg treated patients (0.27 (95% CI 0.16 to 0.46)). Across treatment arms, the crude incidence of non-TB OIs among all tofacitinib users was higher among glucocorticoid users (0.36 (95% CI 0.22 to 0.58)) than non-users (0.13 (95% CI 0.06 to 0.30)). Rates were similar in those aged ≥65 and <65 years (0.21 (95% CI 0.07 to 0.66) vs 0.26 (95% CI 0.17 to 0.40)).

### Tuberculosis

There were 26 cases of active TB reported by investigators, all within tofacitinib-treated individuals from phase III (n=9 cases) and LTE studies (n=17 cases). The median time between tofacitinib start and TB diagnosis was 64 weeks (range, 15–161 weeks). Fifteen (58%) cases involved extrapulmonary infection sites. Few were culture-confirmed, and most (20/26, 77%) occurred in those taking tofacitinib 10 mg twice daily (table 2). All but two cases had negative screening results at entry. The two cases with positive screening results at baseline had a history of receiving an adequate course of therapy for active TB or LTBI in the past (one each). These patients, given the adequate treatment history, were allowed into the trial without isoniazid treatment, per protocol. The TB rate varied according to regional background IR: 21 cases (81%) occurred in countries with high background TB IR (characteristics of the 26 reported active TB cases from phase II, III and LTE studies of tofacitinib are presented in online supplementary file 3).

**Table 2:** TB IRs for tofacitinib patients by background country IRs* (phase II, III and LTE studies)

	TB cases with tofacitinib (n)	Tofacitinib exposure (patient-years)	Crude TB IR † (95% CI)
Low‡ (0.01)	1	4852.3	0.02 (0.003 to 0.15)
Medium§ (≥0.01 and ≤0.05)	4	5020.5	0.08 (0.03 to 0.21)
High¶ (>0.05)	21	2791.1	0.75 (0.49 to 1.15)

*TB background country IR categories from WHO, 2011 report for year 2010.^26^

†Crude incidence calculated TB cases per 100 patient-years.

**‡Low TB incidence region (total study enrolment, n=2213);** the USA (n=1098), Czech Republic (n=378), Germany (n=238), Slovakia (n=126), Australia (n=114), Canada (n=103), Austria (n=36), Italy (n=28), Sweden (n=17), Finland (n=16), Greece (n=15), Belgium (n=13), France (n=10), Denmark (n=9), New Zealand (n=9) Ireland (n=3).

**§Medium TB incidence region (total study enrolment, n=2132);** Japan (n=556), Brazil (n=306), Mexico (n=259), Poland (n=254), Chile (n=174), Bulgaria (n=169), Spain (n=128), Colombia (n=120), Argentina (n=61), Croatia (n=27), Hungary (n=27), the UK (n=17), Costa Rica (n=16), Venezuela (n=7), Bosnia-Herzegovina (n=6), Turkey (n=5).

**¶High TB IR (total study enrolment, n=1326);** Korea (n=284), Ukraine (n=227), China (n=213), India (n=194), Russia (n=149), Thailand (n=63), Philippines (n=61), Malaysia (n=46), Taiwan (n=36), Dominican-Republic (n=27), Peru (n=14), Romania (n=12).

IR, incidence rate; LTE, long-term extension; TB, tuberculosis.

In phase III studies, crude IR of TB among all tofacitinib users was 0.27 (95% CI 0.14 to 0.54) and all cases occurred in the 10 mg twice daily group (crude incidence, 0.53 (95% CI 0.27 to 1.07)). IRs were similar between those aged <65 years (0.54 (95% CI 0.26 to 1.13)) and those ≥65 years (0.51 (95% CI 0.07 to 3.63)), among those with and without baseline glucocorticoid use (0.50 (95% CI 0.19 to 1.33) vs 0.57 (95% CI 0.22 to 1.53)), but trended higher in males (1.26 (95% CI 0.41 to 3.90)) vs females (0.40 (95% CI 0.17 to 0.95)). Within LTE studies, cases occurred at similar incidence in both 5 and 10 mg twice daily arms (0.15 (95% CI 0.07 to 0.33) vs 0.21 (95% CI 0.12 to 0.38)). When examining IRs across dosage arms within the LTE experience, we observed higher incidence in patients aged ≥65 years (0.28 (95% CI 0.11 to 0.75)) compared with those <65 years (0.17 (95% CI 0.10 to 0.29)). Rates were similar among patients using glucocorticoids (0.19 (95% CI 0.10 to 0.37)) and those not using glucocorticoids (0.18 (95% CI 0.09 to 0.36)).

### TB screening results

In phase II studies, patients screening positive for TB were disallowed entry to the trial. In phase III studies, 4088 underwent QFT-IT, of whom 217 (5.3%) had positive results. In total, 305 patients underwent TST testing, of whom 28 (9.2%) had positive results. Some patients lacked screening results. Presumably these patients had a history of TB (treated or untreated) elicited by site investigators; however, this information was not reliably recorded by investigators. In total, 286 patients were reported to have untreated LTBI upon screening. All of these patients completed 1 month of isoniazid prior to starting study drug or placebo, and all completed 9-month regimens of isoniazid; none developed active TB. While tofacitinib-treated patients using isoniazid were slightly more likely to develop small elevations in liver enzymes during therapy, they were no more likely to develop significant liver transaminases elevation (>3× upper limit of normal) during therapy than tofacitinib-treated patients not using isoniazid (table 3).

**Table 3:** Patients in phase III studies who experienced ALT elevations according to study exposure group and isoniazid usage

	>1×ULN n (%)	>3×ULN n (%)	>5×ULN n (%)	>10×ULN n (%)
Isoniazid/tofacitinib* (n=263)	58 (22.1%)	4 (1.5%)	1 (0.4%)	0
No isoniazid/tofacitinib* (n=3614)	550 (15.2%)	35 (1.0%)	7 (0.2%)	2 (<0.1%)
Isoniazid/adalimumab (n=15)	3 (20%)	0	0	0
No isoniazid/adalimumab (n=189)	24 (12.7%)	2 (1.1%)	1 (0.5%)	0
Isoniazid/methotrexate (n=8)	3 (37.5%)	1 (12.5%)	0	0
No isoniazid methotrexate (n=178)	19 (10.7%)	0	0	0

In phase III trials, patients diagnosed with LTBI were allowed trial entry 1 month after starting isoniazid therapy.

*Includes patients who were randomised to tofacitinib at study start and those randomised to placebo who later took tofacitinib during their isoniazid treatment course.

ALT, alanine aminotransferase; LTBI, latent tuberculosis infection; ULN, upper limit of normal.

## Discussion

We have conducted the largest analyses to date examining the risk of OIs in the context of JAK inhibition by tofacitinib. Within the global RA clinical development programme, TB was the most commonly reported OI but was rare in regions of low and medium background TB prevalence. In phase III trials, the incidence of OIs was higher in patients using tofacitinib 10 mg twice daily dosing compared with tofacitinib 5 mg twice daily dosing, and overall most TB cases occurred in patients using the higher dose. Importantly, our data suggest the utility of screening for TB prior to tofacitinib start, and the ability to successfully treat LTBI with isoniazid during tofacitinib therapy.

Patients with RA are known to be at higher risk for OIs, in part due to their therapies. For anti-TNF therapies, this increased risk has been most clearly described for those pathogens that rely upon granulomatous immunity, with TB the best studied example and generally the most common OI (other than routine/uncomplicated herpes zoster) reported in that setting.^27–30^ Further, no matter the immunosuppressive therapy, the risk of TB varies directly with background TB rate in the underlying population, and for those starting TNF blockade, screening and treatment of LTBI effectively reduces the risk of subsequent TB.^31^ In this regard, our experience within the tofacitinib programme was similar. TB rates among tofacitinib-treated individuals varied according to the underlying population's background TB prevalence with higher rates observed in regions of high background TB prevalence. Within North America and Western Europe, we observed TB rates 5–10 times higher than background populations and similar to those described in recent population-based studies assessing the risks of anti-TNF therapies. Early population-based studies conducted in low-prevalence TB regions, before widespread introduction of TB screening prior to biological initiation, reported rates of anti-TNF-associated TB 5–20 times higher than background general populations.^32^ ^33^ The most recent observational studies conducted in the USA and Western Europe, during time periods where TB screening prior to biological start was presumably widespread, have documented similar to lower risk estimates associated with anti-TNF therapy with rates <200 per 100 000 patient-years.^34–36^ This includes British and French registry studies for TB where rates were similar (0.095–0.12, respectively). In general, it is difficult to compare rates between studies conducted in regions where the background risk of TB may vary. The comparative risk between biologics and tofacitinib, and the potential risk modification of steroids, is not clear and deserves further population-based study.

Screening and treatment for LTBI prior to biological therapy has been shown to effectively reduce subsequent TB rates.^31^ Our data also suggest a benefit of starting antimicrobial treatment in patients diagnosed with LTBI prior to tofacitinib start. All but two of the TB cases reported in this study occurred in individuals with negative screening test results at baseline. This is not dissimilar to other clinical trial experiences with anti-TNF therapy,^37^ and it suggests the likelihood that some patients have false-negative screening test results at baseline. False-negative TST or Interferon-Gamma Release Assay results are more common in immunosuppressed and older patients,^38^ and it is possible that a strategy that uses both tests, rather than a single test as reported herein, could improve the sensitivity (and potentially decrease the specificity) of screening prior to starting biologics or tofacitinib.^39^ It is also possible that some of the observed TB cases were instances of newly acquired infection during the trial, given that nearly all the cases occurred in regions of high TB endemicity where exposure would be more likely. The long time between tofacitinib start and TB diagnosis for most of the observed cases, and their negative baseline screening results, lends further support to this idea.^40^ Lastly, it is possible that some of the TB cases were not true cases. We attempted to use US Centers for Disease Control and Prevention case criteria for TB in order to confirm them, but most case reports were lacking in follow-up information, making such adjudication impossible. Only 8 of the 26 cases had microbiological confirmation, and many of the patients were worked-up and diagnosed only by positive acid-fast bacilli smear and no culture was performed. In some of these cases, particularly the pulmonary cases, infection due to NTM was possible and also consistent with the negative screening test results for TB at baseline. There were at least two pulmonary NTM cases reported within the development programme. In regions of low TB prevalence, such as North America and Western Europe, NTM disease is more common than TB,^34^ ^41^ and in fact in our study only two cases of TB occurred in North America and Europe.

Both fungal and viral OIs were also identified in this study. While our data suggest an increased risk of such infections in patients using tofacitinib, it is unclear whether this risk is any different than that associated with biological therapy. It is difficult to compare overall rates of OIs between various studies due to heterogeneity in methods, and particularly due to differences in defining which OIs are considered ‘opportunistic’. In population-based studies from France, Britain and the USA, reported rates vary between 152 and 3000 per 100 000, with the wide variance due to differences in case-finding methodology and OI definitions (specifically some studies included herpes zoster as an OI and others did not).^27–30^ ^42–47^ We have included only complicated cases of herpes zoster within this current analysis and have recently reported uncomplicated herpes zoster rates associated with the development programme.^48^ In that analysis, zoster rates were observed to be elevated with tofacitinib and to vary considerably according to enrolment region, with rates in some Asian countries approximately double those seen in Western Europe or North America. Intracellular infections with *Pneumocystis, Cryptococcus* and CMV were also observed in the development programme. No cases of endemic mycotic infections (histoplasmosis, coccidioidomycosis and blastomycosis) were observed, but presumably few patients were enrolled within regions where these organisms are endemic.

A biological mechanism for how tofacitinib could increase the risk of TB or other intracellular infections is not yet clear. It could theoretically inhibit the development and/or maintenance of pathogen-specific memory T cells by inhibiting the intracellular signalling of IL-12, interferon (IFN)-γ and other relevant cytokines.^49^ An increased risk for serious TB disease has been documented with mutations affecting IL-12, IFN-γ and STAT1 pathways.^50^ Therefore, it is possible that down-modulation of these pathways by JAK inhibition could diminish the ability of macrophages to contain infections such as TB.^51^ Further, it is likely that JAK inhibition diminishes type 1 (IFN-α and IFN-β) and type 2 (IFN-γ) antiviral responses,^52^ both of which signal via the JAK1 receptor. This could explain the spectrum of viral infections observed in the development programme, and such hypotheses deserve further testing.

Our experience suggests that patients can use isoniazid therapy during tofacitinib therapy with good tolerance and apparent efficacy in TB prevention. None of the >200 patients treated in this fashion developed clinically significant hepatitis, all completed isoniazid therapy and none developed active TB. Importantly, it should be noted that a drug–drug interaction exists between rifampin and tofacitinib such that tofacitinib could be less effective during rifampin therapy due to an 80% reduction in bioavailability of tofacitinib.^10^ For this reason, isoniazid should remain the drug of choice when treating LTBI during tofacitinib therapy, and periodic liver function testing should be conducted during such therapy in accordance with Centers for Disease Control and Prevention guidance.^53^

In summary, we observed an increased risk of OIs among patients with RA using tofacitinib, although they occur rarely and are less frequent in those treated with 5 mg twice daily. TB was the most common OI reported in this setting, but remained rare in regions of low TB prevalence. As with biological therapy, screening and treating for LTBI should be employed prior to starting tofacitinib, and long-term population-based studies are necessary to better understand the comparative risk of tofacitinib with other DMARD therapies.

## Supplementary Material

Web supplement

## References

[1] Novosad SA, Winthrop KL Beyond tumor necrosis factor inhibition: the expanding pipeline of biologic therapies for inflammatory diseases and their associated infectious sequelae. Clin Infect Dis 2014;58:1587–98. 10.1093/cid/ciu104 24585557

[2] Ramiro S, Gaujoux-Viala C, Nam JL, et al Safety of synthetic and biological DMARDs: a systematic literature review informing the 2013 update of the EULAR recommendations for management of rheumatoid arthritis. Ann Rheum Dis 2014;73:529–35. 10.1136/annrheumdis-2013-204575 24401994

[3] Jick SS, Lieberman ES, Rahman MU, et al Glucocorticoid use, other associated factors, and the risk of tuberculosis. Arthritis Rheum 2006;55:19–26. 10.1002/art.21705 16463407

[4] Winthrop KL, Iseman M Bedfellows: mycobacteria and rheumatoid arthritis in the era of biologic therapy. Nat Rev Rheumatol 2013;9:524–31. 10.1038/nrrheum.2013.82 23797309

[5] Deepe GS Jr Modulation of infection with Histoplasma capsulatum by inhibition of tumor necrosis factor-alpha activity. Clin Infect Dis 2005;41 (Suppl 3):S204–7. 10.1086/429999 15983901

[6] Wallis RS, Broder M, Wong J, et al Granulomatous infections due to tumor necrosis factor blockade: correction. Clin Infect Dis 2004;39:1254–5. 10.1086/424455 15486857

[7] Wallis RS, Broder MS, Wong JY, et al Granulomatous infectious diseases associated with tumor necrosis factor antagonists. Clin Infect Dis 2004;38:1261–5. 10.1086/383317 15127338

[8] Marino S, Sud D, Plessner H, et al Differences in reactivation of tuberculosis induced from anti-TNF treatments are based on bioavailability in granulomatous tissue. PLoS Comput Biol 2007;3:1909–24. 10.1371/journal.pcbi.0030194 17953477 PMC2041971

[9] Winthrop KL, Chiller T Preventing and treating biologic-associated opportunistic infections. Nat Rev Rheumatol 2009;5:405–10. 10.1038/nrrheum.2009.105 19568254

[10] Pfizer Inc. Xeljanz prescribing information. http://labeling.pfizer.com/ShowLabeling.aspx?id=959 (accessed 22 Jul 2014).

[11] Meyer DM, Jesson MI, Li X, et al Anti-inflammatory activity and neutrophil reductions mediated by the JAK1/JAK3 inhibitor, CP-690,550, in rat adjuvant-induced arthritis. J Inflamm (Lond) 2010;7:41 10.1186/1476-9255-7-41 20701804 PMC2928212

[12] Kremer JM, Cohen S, Wilkinson BE, et al A phase IIb dose-ranging study of the oral JAK inhibitor tofacitinib (CP-690,550) versus placebo in combination with background methotrexate in patients with active rheumatoid arthritis and an inadequate response to methotrexate alone. Arthritis Rheum 2012;64:970–81. 10.1002/art.33419 22006202

[13] Kremer JM, Bloom BJ, Breedveld FC, et al The safety and efficacy of a JAK inhibitor in patients with active rheumatoid arthritis: Results of a double-blind, placebo-controlled phase IIa trial of three dosage levels of CP-690,550 versus placebo. Arthritis Rheum 2009;60:1895–905. 10.1002/art.24567 19565475

[14] Fleischmann R, Cutolo M, Genovese MC, et al Phase IIb dose-ranging study of the oral JAK inhibitor tofacitinib (CP-690,550) or adalimumab monotherapy versus placebo in patients with active rheumatoid arthritis with an inadequate response to disease-modifying antirheumatic drugs. Arthritis Rheum 2012;64:617–29. 10.1002/art.33383 21952978

[15] Tanaka Y, Suzuki M, Nakamura H, et al Phase II study of tofacitinib (CP-690,550) combined with methotrexate in patients with rheumatoid arthritis and an inadequate response to methotrexate. Arthritis Care Res (Hoboken) 2011;63:1150–8. 10.1002/acr.20494 21584942

[16] Tanaka Y, Takeuchi T, Yamanaka H, et al Efficacy and safety of tofacitinib as monotherapy in Japanese patients with active rheumatoid arthritis: a 12-week, randomized, phase 2 study. Mod Rheumatol 2015;25:514–21. 10.3109/14397595.2014.995875 25496464 PMC4819568

[17] McInnes IB, Kim HY, Lee SH, et al Open-label tofacitinib and double-blind atorvastatin in rheumatoid arthritis patients: a randomised study. Ann Rheum Dis 2014;73:124–31. 10.1136/annrheumdis-2012-202442 23482473

[18] Burmester GR, Panaccione R, Gordon KB, et al Adalimumab: long-term safety in 23 458 patients from global clinical trials in rheumatoid arthritis, juvenile idiopathic arthritis, ankylosing spondylitis, psoriatic arthritis, psoriasis and Crohn's disease. Ann Rheum Dis 2013;72:517–24. 10.1136/annrheumdis-2011-201244 22562972 PMC3595151

[19] Kremer J, Li ZG, Hall S, et al Tofacitinib in Combination with Nonbiologic DMARDs in Patients with Active Rheumatoid Arthritis: A Randomized Trial. Ann Intern Med 2013;159:253–61. 10.7326/0003-4819-159-4-201308200-00006 24026258

[20] Fleischmann R, Kremer J, Cush J, et al Placebo-controlled trial of tofacitinib monotherapy in rheumatoid arthritis. N Engl J Med 2012;367:495–507. 10.1056/NEJMoa1109071 22873530

[21] van der Heijde D, Tanaka Y, Fleischmann R, et al Tofacitinib (CP-690,550) in patients with rheumatoid arthritis receiving methotrexate: twelve-month data from a twenty-four-month phase III randomized radiographic study. Arthritis Rheum 2013;65:559–70. 10.1002/art.37816 23348607

[22] van Vollenhoven RF, Fleischmann R, Cohen S, et al Tofacitinib or adalimumab versus placebo in rheumatoid arthritis. N Engl J Med 2012;367:508–19. 10.1056/NEJMoa1112072 22873531

[23] Lee EB, Fleischmann R, Hall S, et al Tofacitinib versus Methotrexate in Rheumatoid Arthritis. N Engl J Med 2014;370:2377–86. 10.1056/NEJMoa1310476 24941177

[24] Wollenhaupt J, Silverfield J, Lee EB, et al Tofacitinib, an oral janus kinase inhibitor, in the treatment of rheumatoid arthritis: open-label, long-term extension safety and efficacy up to 48 months [abstract]. Arthritis Rheum 2012;64 (Suppl 10):S548.

[25] Yamanaka H, Tanaka Y, Takeuchi T, et al An oral janus kinase inhibitor, as monotherapy or with background methotrexate in japanese patients with rheumatoid arthritis: a phase 2/3 long-term extension study [abstract]. Arthritis Rheum 2011;63:S473 10.1002/acr.20567 PMC4730592 26818974

[26] World Health Organization. Global tuberculosis control: WHO report. 2011 http://whqlibdoc.who.int/publications/2011/9789241564380_eng.pdf (accessed 17 Oct 2014).

[27] Greenberg JD, Reed G, Kremer JM, et al Association of methotrexate and tumour necrosis factor antagonists with risk of infectious outcomes including opportunistic infections in the CORRONA registry. Ann Rheum Dis 2010;69:380–6. 10.1136/ard.2008.089276 19359261 PMC2861900

[28] Dixon WG, Watson K, Lunt M, et al Rates of serious infection, including site-specific and bacterial intracellular infection, in rheumatoid arthritis patients receiving anti-tumor necrosis factor therapy: results from the British Society for Rheumatology Biologics Register. Arthritis Rheum 2006;54:2368–76. 10.1002/art.21978 16868999

[29] Salmon-Ceron D, Tubach F, Lortholary O, et al Drug-specific risk of non-tuberculosis opportunistic infections in patients receiving anti-TNF therapy reported to the 3-year prospective French RATIO registry. Ann Rheum Dis 2011;70:616–23. 10.1136/ard.2010.137422 21177290

[30] Winthrop KL Infections and biologic therapy in rheumatoid arthritis: our changing understanding of risk and prevention. Rheum Dis Clin North Am 2012;38:727–45. 10.1016/j.rdc.2012.08.019 23137579

[31] Carmona L, Gomez-Reino JJ, Rodriguez-Valverde V, et al Effectiveness of recommendations to prevent reactivation of latent tuberculosis infection in patients treated with tumor necrosis factor antagonists. Arthritis Rheum 2005;52:1766–72. 10.1002/art.21043 15934089

[32] Askling J, Fored CM, Brandt L, et al Risk and case characteristics of tuberculosis in rheumatoid arthritis associated with tumor necrosis factor antagonists in Sweden. Arthritis Rheum 2005;52:1986–92. 10.1002/art.21137 15986370

[33] Wolfe F, Michaud K, Anderson J, et al Tuberculosis infection in patients with rheumatoid arthritis and the effect of infliximab therapy. Arthritis Rheum 2004;50:372–9. 10.1002/art.20009 14872478

[34] Winthrop K, Baxter R, Liu L, et al Mycobacterial diseases and antitumour necrosis factor therapy in USA. Ann Rheum Dis 2013;72:37–42. 10.1136/annrheumdis-2011-200690 22523429

[35] Arkema EV, Jonsson J, Baecklund E, et al Are patients with rheumatoid arthritis still at an increased risk of tuberculosis and what is the role of biological treatments? Ann Rheum Dis 2015;74:1212–17. 10.1136/annrheumdis-2013-204960 24608401

[36] Tubach F, Salmon D, Ravaud P, et al Risk of tuberculosis is higher with anti-tumor necrosis factor monoclonal antibody therapy than with soluble tumor necrosis factor receptor therapy: the three-year prospective French Research Axed on Tolerance of Biotherapies registry. Arthritis Rheum 2009;60:1884–94. 10.1002/art.24632 19565495 PMC2921546

[37] Hsia EC, Cush JJ, Matteson EL, et al Comprehensive tuberculosis screening program in patients with inflammatory arthritides treated with golimumab, a human anti-tumor necrosis factor antibody, in Phase III clinical trials. Arthritis Care Res (Hoboken) 2013;65:309–13. 10.1002/acr.21788 22782640

[38] Acevedo-Vasquez E, Ponce de Leon D, Gamboa-Cardena R Latent infection and tuberculosis disease in rheumatoid arthritis patients. Rheum Dis Clin North Am 2009;35:163–81. 10.1016/j.rdc.2009.03.008 19481003

[39] Winthrop KL, Weinblatt ME, Daley CL You can't always get what you want, but if you try sometimes (with two tests—TST and IGRA—for tuberculosis) you get what you need. Ann Rheum Dis 2012;71:1757–60. 10.1136/annrheumdis-2012-201979 22975752

[40] Wallis RS Mathematical modeling of the cause of tuberculosis during tumor necrosis factor blockade. Arthritis Rheum 2008;58:947–52. 10.1002/art.23285 18383389

[41] Winthrop KL, Yamashita S, Beekmann SE, et al Mycobacterial and other serious infections in patients receiving anti-tumor necrosis factor and other newly approved biologic therapies: case finding through the Emerging Infections Network. Clin Infect Dis 2008;46:1738–40. 10.1086/587989 18419421

[42] Dixon WG, Hyrich KL, Watson KD, et al Drug-specific risk of tuberculosis in patients with rheumatoid arthritis treated with anti-TNF therapy: results from the British Society for Rheumatology Biologics Register (BSRBR). Ann Rheum Dis 2010;69:522–8. 10.1136/ard.2009.118935 19854715 PMC2927681

[43] Winthrop KL, Baxter R, Liu L, et al The reliability of diagnostic coding and laboratory data to identify tuberculosis and nontuberculous mycobacterial disease among rheumatoid arthritis patients using anti-tumor necrosis factor therapy. Pharmacoepidemiol Drug Saf 2011;20:229–35. 10.1002/pds.2049 21351303 PMC4094092

[44] Winthrop KL, Calabrese LH Let the fog be lifted: screening for hepatitis B virus before biological therapy. Ann Rheum Dis 2011;70:1701–3. 10.1136/annrheumdis-2011-200163 21893581

[45] McDonald JR, Zeringue AL, Caplan L, et al Herpes zoster risk factors in a national cohort of veterans with rheumatoid arthritis. Clin Infect Dis 2009;48:1364–71. 10.1086/598331 19368499 PMC2743911

[46] Strangfeld A, Eveslage M, Schneider M, et al Treatment benefit or survival of the fittest: what drives the time-dependent decrease in serious infection rates under TNF inhibition and what does this imply for the individual patient? Ann Rheum Dis 2011;70:1914–20. 10.1136/ard.2011.151043 21791449 PMC3184240

[47] Galloway JB, Hyrich KL, Mercer LK, et al Risk of septic arthritis in patients with rheumatoid arthritis and the effect of anti-TNF therapy: results from the British Society for Rheumatology Biologics Register. Ann Rheum Dis 2011;70:1810–14. 10.1136/ard.2011.152769 21784730 PMC3168332

[48] Winthrop KL, Yamanaka H, Valdez H, et al Herpes zoster and tofacitinib therapy in patients with rheumatoid arthritis. Arthritis Rheumatol 2014;66:2675–84. 10.1002/art.38745 24943354 PMC4285807

[49] Paniagua R, Si MS, Flores MG, et al Effects of JAK3 inhibition with CP-690,550 on immune cell populations and their functions in nonhuman primate recipients of kidney allografts. Transplantation 2005;80:1283–92. 10.1097/01.tp.0000177643.05739.cd 16314797

[50] Abel L, El-Baghdadi J, Bousfiha AA, et al Human genetics of tuberculosis: a long and winding road. Philos Trans R Soc Lond B Biol Sci 2014;369:20130428 10.1098/rstb.2013.0428 24821915 PMC4024222

[51] Maiga M, Lun S, Guo H, et al Risk of tuberculosis reactivation with tofacitinib (CP-690550). J Infect Dis 2012;205:1705–8. 10.1093/infdis/jis269 22474037 PMC3415851

[52] Malmgaard L Induction and regulation of IFNs during viral infections. J Interferon Cytokine Res 2004;24:439–54. 10.1089/1079990041689665 15320958

[53] American Thoracic Society, Centers for Disease Control and Prevention. Targeted tuberculin testing and treatment of latent tuberculosis infection. MMWR 2000;49(RR06):1–54. 10881762

