# Supplementary material for: Tuberculosis and other opportunistic infections in tofacitinib-treated patients with rheumatoid arthritis
Source: Ann Rheum Dis. 2015 Aug 28;75(6):1133–8. doi: 10.1136/annrheumdis-2015-207319 (PMC4893093; doi:10.1136/annrheumdis-2015-207319)
Supplement: Web supplement [file annrheumdis-2015-207319-s1.pdf]

Supplementary Appendix

Tuberculosis and other opportunistic infections in tofacitinib-treated rheumatoid arthritis patients

[1]Winthrop KL, [2]Park S-H, [3]Gul A, [4]Cardiel M, [5]Gomez-Reino JJ, [6]Tanaka Y, [7]Kwok K, [7]Lukic T, [8]Mortenson E, [7]Ponce de Leon D, [7]Riese R, [9]Valdez H

[1]Oregon Health and Science University, Portland, Oregon, USA;

[2]Seoul St. Mary's Hospital, The Catholic University of Korea, South Korea;

[3]Istanbul Faculty of Medicine, Istanbul University, Istanbul, Turkey;

[4]Centro de Investigación Clínica de Morelia SC, Morelia, Mexico;

[5]Hospital Clinico Universitario de Santiago, Santiago de Compostela, Spain;

[6]University of Occupational and Environmental Health, Kitakyushu, Japan;

[7]Pfizer Inc, Groton, CT, USA.

[8]Pfizer Inc, New York, NY, USA

[9]Pfizer Inc, Collegeville, Pennsylvania, USA.

Supplementary file 1 – MedDRA search terms for case finding

Opportunistic Infections (ADJUDICATED)

1. Treated Subject
2. Event started on or after start of study med treatment
3. Event is one of these preferred terms for P2P3 and pid^= ‘A3921064 1037 10371006’:
    a. ESOPHAGEAL CANDIDIASIS
    b. SIALOADENITIS
    c. PNEUMOCYSTIS JIROVECI PNEUMONIA
    d. CMV VIREMIA
    e. CYTOMEGALOVIRUS VIRAEMIA
    f. PNEUMONIA CRYPTOCOCCAL
    g. HERPES ZOSTER DISSEMINATED
    h. LYMPH NODE TUBERCULOSIS
    i. TUBERCULOUS PLEURISY
    j. PULMONARY TUBERCULOSIS
4. Event is one of these preferred terms for LTE:
    a. Cytomegalovirus Infection (10011831)
    b. Encephalitis (10014581)
    c. Hepatitis acute (10019727)
    d. Meningitis cryptococcal (10027209)
    e. Oesophageal candidiasis (10030154)
    f. Mycobacterium avium complex infection (10058806)
    g. Atypical mycobacterial infection (10061663)
    h. Pneumocystis jirovecii pneumonia (10064108)
    i. Disseminated tuberculosis (10013453)
    j. Tuberculosis (10044755)
    k. Bone tuberculosis (10056377)
    l. Tuberculosis of central nervous system (10061391)
    m. Pulmonary tuberculosis (10037440)
    n. Encephalitis (10014581)

o. Hepatitis acute (10019727)
p. Pneumonia (10035664) and pid in A3921024 1145 11451015 or A3921041 1005 10051007

Code:

P2P3: if factdat^=. and aefdate>=factdat and upcase(preftext) in ('OESOPHAGEAL CANDIDIASIS', 'SIALOADENITIS', 'PNEUMOCYSTIS JIROVECI PNEUMONIA', 'CMV VIREMIA', 'CYTOMEGALOVIRUS VIRAEMIA', 'PNEUMONIA CRYPTOCOCCAL', 'HERPES ZOSTER DISSEMINATED', 'LYMPH NODE TUBERCULOSIS', 'TUBERCULOUS PLEURISY', 'PULMONARY TUBERCULOSIS') and upcase(pid) not in ("A3921064 1037 10371006")

LTE: if factdat^=. and aefdate>=factdat and (prefcode in (10011831, 10014581, 10019727, 10027209, 10030154, 10058806, 10061663, 10064108, 10013453, 10044755, 10056377, 10061391, 10037440, 10014581, 10019727) or (PID in ("A3921024 1145 11451015","A3921041 1005 10051007") and prefcode in (10035664)))

NOTE – aeser=1 can be added for Serious Opportunistic Infections

Supplementary file 2 – Crude incidence rate of OIs (TB and non-TB OIs combined) across various data within the tofacitinib developmental program[a] for rheumatoid arthritis

**Factor**	**Strata**	**OI cases (n)**	**Crude incidence rate (95% CI)[b]**
Age	<65 years	48	0.44 (0.33, 0.58)
	≥65 years	10	0.58 (0.31, 1.07)
Sex	Male	6	0.28 (0.13, 0.63)
	Female	52	0.49 (0.38, 0.65)
Glucocorticoids	No	21	0.38 (0.25, 0.58)
	Yes	37	0.52 (0.38, 0.72)
TB region[c]	Low	7	0.14 (0.07, 0.30)
	Medium	23	0.46 (0.31, 0.69)
	High	28	1.01 (0.69, 1.46)

[a]Phase 2, 3 and long-term extension study data

[b]per 100 patient-years of exposure

[c]Based on WHO background incidence

CI, confidence interval; OI, opportunistic infection; TB, tuberculosis

Supplementary file 3 – Characteristics of the 26 reported active TB cases from Phase 2, 3, and LTE studies of tofacitinib

Tofacitinib dose	Country	Age at time of TB onset	Sex	Days to onset	Meets CDC TB criteria[a]	TB location	TB screening	Bacteriology	Methotrexate	Prednisone	Other
10 mg BID	Thailand	43	M	104	Yes	Disseminated	QFT negative	Sputum TB PCR+	No	No	Pericardial, pulmonary, lymph node involvement
10 mg BID	India	48	M	152	Yes	Pulmonary	QFT indeterminate and TST negative (0 mm)	None	No	No	Pleural effusion
5 mg BID	USA	73	F	196	Yes	Disseminated TB	QFT negative	Positive DNA M Tb and M avium	No	Yes	None
10 mg BID	Thailand	51	F	201	No	Pulmonary	QFT positive; history of treated TB	Positive AFB sputum smear	Yes	No	No culture information; history of infected bronchiectasis
5 mg BID	Mexico	42	F	240	Yes	Disseminated TB	TST negative (0 mm)	Positive AFB smear from gastric aspirate	Yes	No	Negative culture and QuantiFERON® during event
10 mg BID	India	43	F	269	Yes	Supraclavicular node	QFT negative	Lymph node biopsy "positive for TB"	Yes	No	None
10 mg BID	Philippines	67	F	290	No	Pulmonary	QFT negative	Caseating granuloma on lung biopsy	Unknown	Unknown	Diabetes
10 mg BID	Korea	63	M	305	Yes	Pulmonary	QFT negative	Positive AFB sputum smear	Yes	Yes	No culture report

Tofacitinib dose	Country	Age at time of TB onset	Sex	Days to onset	Meets CDC TB criteria[a]	TB location	TB screening	Bacteriology	Methotrexate	Predniso ne	Other
10 mg BID	India	53	F	325	Yes	Bone	QFT negative	None	No	No	Necrotizing granuloma on bone biopsy. No cultures
10 mg BID	China	51	M	325	Yes	Pulmonary	QFT negative	Positive AFB sputum smear	Yes	No	No culture, also leflunomide
10 mg BID	Philippine s	73	F	366	Yes	Pulmonary	QFT negative	None	Yes	No	Pleural effusion
5 mg BID then 10 mg BID	Philippine s	71	F	421	No	Pulmonary	QFT negative	None	Yes	No	None
10 mg BID	Korea	55	F	443	Yes	Pericardial/ pleural	QFT negative	Pleural fluid AFB culture negative	No	No	QFT became positive in context of pleural effusion showing lymphocyte dominant exudates with ADA elevation
10 mg BID	Philippine s	43	F	446	Yes	Pulmonary	QFT indeterminate and PPD negative (0 mm)	Negative AFB sputum smear	No	Yes	None
10 mg BID	Korea	62	F	546	Yes	Disseminated	QFT negative	TB PCR+ lymph node	Yes	Yes	
5 mg BID	China	27	F	588	Yes	Disseminated	QFT negative	none	Yes	Yes	Chest CT with milia, septic knee
5 mg BID	Japan	59	F	618	Yes	Pulmonary	QFT negative	Sputum TB culture positive	No	No	
10 mg BID	Philippines	62	F	778	No	Pulmonary	QFT negative	Unknown	Yes	Yes	
5 mg BID	China	38	F	822	No	Pulmonary/ pleural	QFT negative	Effusion AFB smear negative. No cultures done	Yes	No	CXR with infiltrate

Tofacitinib dose	Country	Age at time of TB onset	Sex	Days to onset	Meets CDC TB criteria[a]	TB location	TB screening	Bacteriology	Methotrexate	Predniso ne	Other
10 mg BID	Spain	64	F	845	Yes	Disseminated	QFT positive	Not reported	No	No	MRI brain c/w TB, milia on chest CT
10 mg BID	Korea	42	F	867	Yes	Lymphadenitis	QFT negative	PCR+ node	Yes	No	
5 mg BID	Korea	61	F	960	Yes	Disseminated TB	QFT negative	Positive DNA M Tb bone aspirate	Yes	No	Encephalitis, pulmonary and spinal involvement
10 mg BID	Ukraine	59	F	964	No	Pulmonary	QFT negative	AFB smear + sputum, culture not done	Yes	Yes	CXR with infiltrate
10 mg BID	India	53	F	1031	No	Pleural	QFT negative	None	Unknown	Unknown	
10 mg BID	Poland	78	F	1086	Yes	Scrofula	QFT negative	TB PCR+ cervical lymph node	Yes	No	
10 mg BID	Philippine s	57	F	1126	No	Pulmonary	QFT negative	AFB sputum smear negative	No	Yes	No cultures done. CXR with infiltrate

[a]US Centers for Disease Control and Prevention[1];

ADA, adenosine deaminase; AFB, acid-fast bacilli; BID, twice daily; CDC, Centers for Disease Control; CT, computed tomography; CXR, chest radiograph; F, female; LTE, long term extension; M, male; MRI, magnetic resonance imaging; PCR, polymerase chain reaction; PPD, purified protein derivative; QFT, QuantiFERON® In-tube; TB, tuberculosis; TST, tuberculin skin test

References

1 Centers for Disease Control and Prevention. Tuberculosis (TB) 2009 Case Definition. Available at: http://wwwn.cdc.gov/NNDSS/script/casedef.aspx?CondYrID=876&DatePub=1/1/2009 12:00:00 AM. Accessed October, 2014.
